# DEPDC1B-mediated USP5 deubiquitination of β-catenin promotes breast cancer metastasis by activating the wnt/β-catenin pathway

**DOI:** 10.1152/ajpcell.00249.2023

**Published:** 2023-08-29

**Authors:** Qingqing Wang, Fengxia Chen, Ningning Yang, Lu Xu, Xiaoyan Yu, Meng Wu, Yunfeng Zhou

**Affiliations:** ^1^Hubei Cancer Clinical Study Center, Hubei Key Laboratory of Tumour Biological Behaviours, Zhongnan Hospital, Wuhan University, Wuhan, Hubei, China; ^2^Department of Radiation Oncology and Medical Oncology, Zhongnan Hospital, Wuhan University, Wuhan, Hubei, China; ^3^Department of Ultrasound, Zhongnan Hospital of Wuhan University, Wuhan, Hubei, China; ^4^Department of Radiation Oncology, The Affiliated Cancer Hospital of Zhengzhou University, Zhengzhou, Henan, China

**Keywords:** breast cancer, DEPDC1B, deubiquitination, USP5, β-catenin

## Abstract

Breast cancer has become the malignant disease with the highest morbidity and mortality among female cancer patients. The prognosis of metastatic breast cancer is very poor, and the therapeutic effects still need to be improved. The molecular mechanism of breast cancer has not been fully clarified. Bioinformatics analysis was used to find the differentially expressed gene that affects the occurrence and development of breast cancer. Furthermore, scratch assays, Transwell assays, immunofluorescence, and Western blotting were used to determine the biological behavior of breast cancer cells affected by DEP domain-containing protein 1B (DEPDC1B). The molecular mechanism was investigated by mass spectrometry analysis, coimmunoprecipitation, and ubiquitin assays. Here, we found that DEPDC1B was highly expressed in breast cancer cells and tissues and was associated with lower overall survival (OS) in patients. We found that DEPDC1B interference significantly inhibited tumor invasion and migration in vitro and tumor metastasis in vivo. Mechanistically, DEPDC1B was first shown to activate the wnt/β-catenin signaling pathway as an oncogene in breast cancer cells. In addition, we also confirmed the interaction between DEPDC1B, ubiquitin-specific protease 5 (USP5), and β-catenin. Then, we found that DEPDC1B mediates the deubiquitination of β-catenin via USP5, which promotes cell invasion and migration. Our findings provide new insights into the carcinogenic mechanism of DEPDC1B, suggesting that DEPDC1B can be considered a potential therapeutic target for breast cancer.

**NEW & NOTEWORTHY** By using bioinformatics analysis and the experimental techniques of cell biology and molecular biology, we found that DEP domain-containing protein 1B (DEPDC1B) can promote the invasion and migration of breast cancer cells and that DEPDC1B mediates the deubiquitination of β-catenin by ubiquitin-specific protease 5 (USP5), thus activating the wnt/β-catenin pathway. Our findings provide new insights into the carcinogenic mechanism of DEPDC1B, suggesting that DEPDC1B can be used as a potential therapeutic target for breast cancer.

## Graphical abstract

**Figure:**
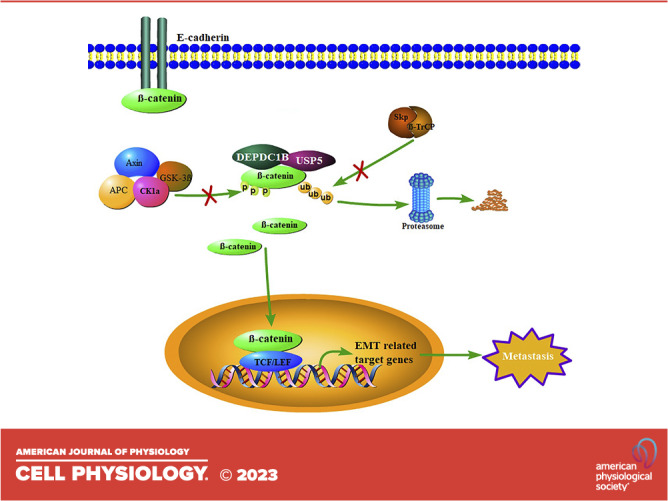

## INTRODUCTION

According to global cancer statistics in 2020, newly diagnosed breast cancer patients ranked first in the world. At the same time, breast cancer deaths accounted for 6.9% of total cancer deaths, ranking fifth among cancer-related deaths (1). Breast cancer is a highly heterogeneous disease. Although for a long time scientific research has been devoted to the occurrence and development of breast cancer, its pathogenesis has not been fully revealed (2). According to statistics, the 5-yr survival rate of patients with breast cancer in situ is as high as 98% (3), whereas metastatic breast cancer cannot be cured. The reason is closely related to the lack of effective treatment targets, resulting in a delay in treatment. As a result, 20–30% of patients cannot obtain clinical benefits because of distant metastasis (4). Therefore, there is an urgent need to determine effective therapeutic targets.

Tumor cells can gain the ability to invade through epithelial-mesenchymal transformation (EMT) and then metastasize distally (5). Deubiquitinating enzymes (DUBs) play an important biological role in tumor metastasis by maintaining the stability of many important proteins. Some DUBs deubiquitinate EMT-related transfer factors, and other expressed genetic regulatory factors mediate the EMT process of tumor cells and promote tumor invasion and migration (6). The ubiquitin-specific protease (USP) family is the largest family of DUBs (7), and there is growing evidence that USP family disorders lead to cancer, especially breast cancer (8, 9). These findings also highlight the possibility that manipulation of deubiquitination events may provide potential targets for cancer treatment.

DEP domain-containing protein 1B (DEPDC1B) is a protein encoded by a gene located on human chromosome 5q12.1. It was screened and identified by Boudreau et al. after Raf-1 siRNA treatment of MDA-MB-231 cells in 2007, and it was found that it is a cell cycle regulatory molecule related to the promotion of ERK phosphorylation and cell proliferation (10). It mainly contains two domains, namely, the DEP domain at the NH_2_ terminus and the RhoGAP domain at the COOH terminus (10). The DEP domain has been found in Dishevelled, EGL-10, and Pleckstrin (11). It can participate in membrane anchoring, signal regulation mediated by G protein-coupled receptors, and activation of the Wnt signaling pathway (12–14). The main function of the RhoGAP domain is to activate the RhoGTPase signaling pathway (15). DEPDC1B plays an important role in a variety of cancers, such as liver cancer (16), chordoma (17), pancreatic cancer (18), and non-small-cell lung cancer (19). It is worth noting that it was reported that DEPDC1B promotes SCUBE3 secretion through competitive binding with ubiquitin ligase CDC16, thus promoting angiogenesis and metastasis of melanoma (20). However, it is not clear whether DEPDC1B plays a role in breast cancer by affecting ubiquitin events.

The purpose of this study was to investigate the role and mechanisms of DEPDC1B in breast cancer cells in vivo and in vitro. Here, we found that DEPDC1B was highly expressed in breast cancer tissues and cells compared with normal tissues and cells. In addition, in breast cancer patients high DEPDC1B levels were associated with shorter overall survival (OS). Importantly, we found that DEPDC1B mediates deubiquitination events in breast cancer cells. Furthermore, we also revealed a novel mechanism in DEPDC1B-mediated USP5 deubiquitination of β-catenin, which led to the activation of the wnt/β-catenin signaling pathway and ultimately promoted breast cancer cell metastasis.

## METHODS

### Microarray Data, Data Processing, and Screening of Differentially Expressed Genes

Breast cancer second-generation sequencing datasets and clinical data were downloaded and obtained from The Cancer Genome Atlas (TCGA) Xena website (https://xenabrowser.net/datapages/). The differentially expressed genes (DEGs) in The Cancer Genome Atlas-Breast cancer (TCGA-BRCA) were analyzed by the limma package (21) in the R programming language, |log fold change (logFC)| > 1 and *P* < 0.05. Then, ggplot2 and pheatmap packages were used to draw volcano maps and heatmaps, respectively. According to the screening results, |logFC| > 3 and *P* < 0.05 were adjusted to find more meaningful DEGs.

### Screening Hub Genes and Analyzing the Correlation between Their Expression and Prognosis

The Search Tool for Retrieval of Interacting Genes (STRING) database (http://string-db.org) was used to construct the interaction network of proteins expressed by DEGs (22), with a combined score > 0.4. Cytoscape 3.8.2 was used for visual analysis of the network (23), Molecular Complex Detection (MCODE) was used to perform module analysis (degree cutoff > 2, node score cutoff > 0.2, K-core > 2 and max dep > 100), and the clustering coefficient of cytoHubba was used to calculate the top 15 hub genes. Then, the Kaplan–Meier plotter (KM plotter, http://kmplot.com) (24, 25) was used to analyze the effect of hub genes (mRNA) on the OS of breast cancer patients, and the most critical hub gene was screened out according to the hazard ratio (HR) value.

The Cancer Cell Line Encyclopedia (CCLE, https://www.broadinstitute.org/ccle) (26, 27) was used to analyze the expression of hub genes in various cancer cell lines. The European Bioinformatics Institute (EMBL-EBI, https://www.ebi.ac.uk) (28) was used to analyze the hub gene expression in breast cancer cells. Oncomine (http://www.oncomine.com) (29) was used to analyze the hub gene expression between breast cancer tissues and normal samples. The hub gene protein expression in breast cancer and normal breast tissues was analyzed by HPA (The Human Protein Atlas, https://www.proteinatlas.org/) (30).

The Gene Expression Profiling Interactive Analysis (GEPIA, http://gepia.cancer-pku.cn) was used to analyze the relationship between hub genes and clinical stage (31). Breast Cancer Gene-Expression Miner v4.5 (Bc-GenExMiner, http://bcgenex.ico.unicancer.fr) was used to analyze the hub gene expression in the breast cancer transcriptional group, clinical correlation, and its effect on prognosis (32).

### Gene Set Enrichment Analysis

The TCGA-BRCA gene expression dataset was downloaded from the Xena web browser (https://xenabrowser.net/datapages/). First, according to the median of DEPDC1B, the TCGA-BRCA dataset was divided into two groups: a high-expression group and a low-expression group for DEPDC1B. The GSEA 4.1.0 software program was downloaded from http://www.broad.mit.edu/gsea/, and the following predefined gene sets were used for GSEA (33): hallmark gene sets, curated gene sets, and gene ontology. Permutation number = 1,000 (*P* < 0.05), false discovery rate (FDR) < 0.05 was considered to be statistically significant.

### Tissue Microarray and Immunohistochemistry Staining

A breast cancer tissue microarray containing 140 cancer sites (HBreD140Su07) and 38 paracancerous sites (HBre-Duc046Sur-02) was obtained from Shanghai Outdo Biotech Company (Shanghai, China). The immunohistochemistry (IHC) staining was performed as described previously (34). Experimentally, dewaxing and rehydration steps were identical to hematoxylin and eosin (HE) staining. We performed antigen repair of the tissue samples with EDTA buffer (pH 8.0) at 100°C for 3 min, washed them with PBS, and soaked them in 3% dioxygen water for 20 min. The purpose for doing so was to block the activity of endogenous peroxidase. The 10% goat serum was prepared, and the sections were kept in it at room temperature for 30 min. Next, primary antibodies were employed to incubate these sections overnight at 4°C. Afterwards, the sections were washed before we incubated them with a secondary antibody for 1 h at room temperature. In addition, the samples were stained with diaminobenzidine (DAB) and fixed with neutral resin. Next, the processed samples were observed under a light microscope before division into five grades (0, no positive cells; 1, <25% positive cells; 2, 26–50% positive cells; 3, 51–75% positive cells; 4, 76–100% positive cells). Furthermore, the staining intensity score was divided into four grades (0, none; 1, weak; 2, moderate; 3, strong). The score for each tissue sample was determined by multiplying the staining intensity and the percentage of stained cells: the higher the score, the higher the expression of indicator molecules. The median expression level was used to determine the critical value of high- and low-expression groups: 85 patients were designated in the low-DEPDC1B group (DEPDC1B expression ≤ 8) and 55 patients were designated in the high-DEPDC1B group (DEPDC1B expression > 8).

### Cell Lines and Cell Culture

The nontumorigenic mammary epithelial cell line MCF-10A, the human breast cancer cell lines MDA-MB-231, MDA-MB-468, MDA-MB-157, and BT-549, and the human embryonic kidney epithelial cell line HEK-293T were purchased from Procell Life Science & Technology Company (Wuhan, China). Another breast cancer cell line, Hs578T, was purchased from the Stem Cell Bank, Chinese Academy of Sciences in Shanghai, China. All cell lines were authenticated by short tandem repeat (STR) analysis, which proved that there was no mycoplasma contamination. MCF-10A cells were cultured in DMEM-F-12 medium (Procell). Hs578T cells were cultured in CM-0114 medium (Procell). MDA-MB-157, MDA-MB-231, MDA-MB-468, and HEK-293T cells were cultured in DMEM (HyClone, United States) containing 10% fetal bovine serum. The BT-549 cells were cultured in RPMI-1640 medium (HyClone) containing 10% fetal bovine serum. All cells were cultured in a humidified atmosphere at 37°C with 5% CO_2_ and 95% air.

### RNA Extraction, Reverse Transcription, and Quantitative Real-Time PCR

These assays were carried out according to a previous description (35). Total RNA was extracted from the cells by TRIzol reagent (Invitrogen, United States). The reverse transcription process for 2 μg of RNA was carried out with the Primescript RT Reagent Kit (Vazyme, Nanjing, China) and quantitative real-time PCR (qRT–PCR) with 2× Cham SYBR Green Supermix (Vazyme). PCR was performed in triplicate with a Bio-Rad iCycler (catalog no. CFX96). To quantify relative gene expression normalized to GAPDH, the $2^{-\Delta \Delta C_{t}}$ method (where C_t_ is threshold cycle) was used. Supplemental Table S1 lists the sequences of all primers used.

### Plasmid Construction and Cell Transfection

The DEPDC1B was amplified and cloned into the Flag-M35 vector by the primer sequences shown in Supplemental Table S1. The DNA sequence was confirmed by sequencing. The small interfering RNA (siRNA) of DEPDC1B and the negative control siRNA were synthesized through GenePharma (Suzhou, China). The siRNA and DEPDC1B plasmid were transfected into breast cancer cells with Lipofectamine 3000 (L3000015; Invitrogen, Waltham, MA) according to the manufacturer’s protocol. After 48 h of transfection, the cells were cultured, and RNA or protein was extracted for subsequent experiments.

### Transwell Assay

For the Transwell assay, cells were seeded in a 24-well plate Transwell chamber system (Corning, United States). Approximately 10^4^ cells per 200 μL of serum-free medium were inoculated in the upper chamber, while in the lower chamber there was medium containing 20% serum. After 24 h of incubation, the cells were fixed and stained as described in the clone formation experiment. Finally, the upper chamber cells were wiped off with cotton swabs and photographed via an optical microscope.

### Wound Healing Assay

Upon 24 h of transfection, the cells were inoculated in a six-well plate and allowed to adhere to a density of >90%. The cells were then scratched and cultured in 2% serum medium and photographed at 0, 12, 24, 48, and 72 h. The percentage of wound healing was calculated via the formula (wound width at 0 h − wound width at the observed time point)/wound width at 0 h × 100.

### Western Blot Assay

Western blotting experiments were conducted as described previously (36). Primary antibodies against DEPDC1B (1:1,000, OM276950; OmnimAbs, United States), N-cadherin (1:1,000, no. 66219-1-lg; Proteintech, China), vimentin (1:1,000, no. 10366-1-AP; Proteintech), SCUBE3 (1:500, no. sc-514696; Santa Cruz, United States), wnt3a (1:1,000, DF6113; Affinity Biosciences, United States), phospho (p-)GSK-3β (ser9) (1:500, AF2016; Affinity Biosciences, United States), GSK-3β (1:700, BF0695; Affinity Biosciences, United States), β-catenin (1:1,000, AF6266; Affinity Biosciences, United States), and GAPDH (1:5,000, no. 60004-1-Ig; Proteintech) were used. The secondary antibodies used included horseradish peroxidase (HRP)-conjugated goat anti-mouse antibody (1:10,000, no. SA00001-1; Proteintech) and HRP-conjugated goat anti-rabbit antibody (1:10,000, no. SA00001-2; Proteintech). The blots were further developed by ECL exposure (Advansta, United States). Then, they were detected with the ChemiDoc XRS+ system (Bio-Rad, Hercules, CA).

### Immunofluorescence Staining and Immunofluorescence Colocalization

Immunofluorescence staining (IF) was conducted as described previously (37). The primary antibodies against E-cadherin (1:100, no. Ab181; Abcam, United States) and β-catenin (1:100, no. Ab22656; Abcam) were incubated overnight at 4°C. The nuclei were stained with DAPI (no. H-1200; Vector, United States) for 5 min. Finally, we employed a Nikon Eclipse Ti-U fluorescence microscope (Diagnostic Instruments Inc., United States) to visualize and image the cells before we quantified them with ImageJ software. MDA-MB-157 was transfected with MYC-USP5, FLAG-DEPDC1B, HA-β-catenin plasmid, and treated like IF, incubation of primary antibodies of HA (1:100, no. 51064-2-Ap; Proteintech), FLAG (1:100, no. T0003; Affinity Biosciences, China), and MYC (1:200, no. 16286-1-Ap; Proteintech) and secondary antibodies coupled with the dyes of 520, 690, 570 (no. OP-001003, AKOYA, United States). The photos were taken by a fluorescence scanner (no. VERSA8, Leica, Germany).

### Mass Spectrometry

Vector and FLAG-DEPDC1B plasmids were transfected into HEK-293T cells, and the extracted proteins were used in mass spectrometry analysis at Shanghai Bioprofile Technology Company Ltd. (Shanghai, China).

### Coimmunoprecipitation

The target antibodies against MYC (no. 16286-1-Ap; Proteintech), FLAG (no. T0003; Affinity Biosciences, China), HA (no. 51064-2-Ap; Proteintech), and IgG (no. PV-6001; ZSGB-BIO, China) at a final concentration of 25 μg/mL were rotated and incubated for 15 min at room temperature and then washed twice with 200 μL of binding buffer. Then, the whole cell lysate was rotated at room temperature for 1 h and washed twice with 200 μL of washing buffer. After mixing with 25 μL of 1× SDS-PAGE loading buffer and then 5 min at 95°C, the supernatant was obtained by centrifugation, and 5 μL was taken for further Western blotting.

### Ubiquitin Assay

MDA-MB-157 cells were transfected with siDEPDC1B, MYC-USP5, and HA-β-catenin, and total protein was extracted after treatment with MG132 for 12 h. Anti-β-catenin was added to the cell lysate and incubated overnight at 4°C. The ubiquitin level of β-catenin was detected by anti-ub (1:100, no. 10201-2-Ap; Proteintech) after immunoprecipitation (IP).

### Generation of DEPDC1B Knockdown Cell Lines

The lentiviral-DEPDC1B-shRNA (Lv-sh-DEPDC1B) and lentiviral-vector-shRNA (Lv-sh-NC) were purchased from GenePharma. The Hs578T stable cell lines were generated through infecting these viruses. After 24 h of transfection, the cells were treated with 2 μg/mL puromycin (Sigma, United States) for a minimum of 7 days to build stable DEPDC1B-knockdown cell lines.

### Xenograft Model of Pulmonary Metastasis

Female BALB/c-nude mice (3 wk old) were purchased from SPF (Beijing) Biotechnology Co., Ltd. (Beijing, China). Then, the animals were classified at random into control and experimental groups, with each group having five mice. The mice were raised in two cages according to group, and the feeding environment was an isolated cage of specific pathogen-free animals whose ambient temperature was 25°C and humidity was maintained at 60%. In addition, plenty of autoclaved food and water were provided. The light environment was consistent with the natural cycle. After 1 wk of adaptive feeding, the mice were in good mental state and weighed 12–16 g. The mice were intravenously injected with 1 × 10^6^ cells/10 μL PBS (Hs578T Lv-sh-NC or Lv-sh-DEPDC1B cells). Two mice who died during the experiment (1 in the experimental group and 1 in the control group) were excluded from the follow-up experiment. Six weeks after vein injection, intraperitoneal injection of pentobarbital sodium was used to anesthetize the mice. The Fusion FX7 Spectra Imaging system (Vilber, France) was used to observe the lung metastasis of breast cancer cells. Finally, HE staining was performed on the lung tissues. Animal experiments were approved by the Institutional Animal Care and Use Committee of Wuhan University.

### Hematoxylin and Eosin Staining

Hematoxylin and eosin (HE) staining was conducted according to previously described protocols (34). Briefly, paraffin sections were dewaxed in xylene (I) and xylene (II) for 5 min and then dehydrated in anhydrous ethanol for 5 min, ethanol (95%, 80%, 70%), and distilled water for 2 min. The sections were then dyed with hematoxylin dye solution for 10 min before being cleaned with water for 30 s. Meanwhile, the slides were dyed with eosin solution for 30 s before they were cleaned with water for 5 min. Then, the slides were dehydrated, xylene transparent. Finally, the slides were sealed with neutral gum and then imaged.

### Statistical Analysis

GraphPad Prism 5 (GraphPad Software, Inc., La Jolla, CA) and SPSS 22.0 software (SPSS, Chicago, IL) were used to analyze data. Based on two-tailed Student’s t test or χ^2^-test, we evaluated the vitality of the differences of the two groups. One-way ANOVA was used for analyzing the data of multiple groups. Meanwhile, the correlation of DEPDC1B expression with overall survival (OS) was evaluated by the log-rank test of the Kaplan–Meier plot. In addition, based on univariate and multivariate Cox regression, the survival data were evaluated. All the data presented as means ± standard deviation (SD) are the statistical results of the three stand-alone experiments. *P* < 0.05 was considered significant.

## RESULTS

### Analysis of DEGs and Hub Gene Identification between Breast Cancer and Normal Tissues

**Figure 1.**
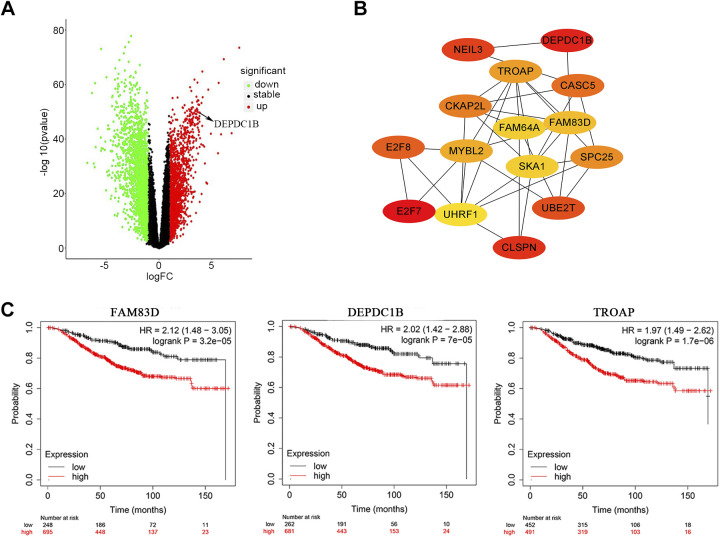
Analysis of differentially expressed genes (DEGs) in The Cancer Genome Atlas-Breast cancer (TCGA-BRCA) and calculation of hub genes. *A*: volcano map, |log fold change (logFC)| > 1, *P* < 0.05. *B*: the cytoHubba plug-in in Cytoscape calculates and analyzes the hub genes in module (Supplemental Fig. S1*C*). The darker the color, the higher the gene score. *C*: the high expression of hub genes was significantly correlated with the shorter survival time of breast cancer patients, and the analysis results were shown from high to low according to hazard ratio (HR); the top 3 are shown here.

Bioinformatics technology to identify key DEGs has been widely used in breast cancer research (38). Through the analysis of BRCA and paracancerous samples in the TCGA database, 4,190 DEGs were obtained (Fig. 1*A*), and 364 DEGs were obtained after improving the screening criteria (Supplemental Table S2, Supplemental Fig. S1*A*). Further PPI network construction (Supplemental Fig. S1*B*), MCODE analysis (Supplemental Table S3), and the most critical module of cytoHubba calculation (Supplemental Fig. S1*C*) yielded the top 15 hub genes (Fig. 1*B*). Through survival analysis, it was found that the high expression of these 15 genes was positively correlated with the shorter OS of breast cancer (Fig. 1*C* and Supplemental Fig. S2). According to the HR value and the order of cytoHubba calculation, it was preliminarily determined that DEPDC1B was the most critical hub gene.

### DEPDC1B Is Upregulated in Breast Cancer Tissues and Cells, and the DEPDC1B Expression Level Is Negatively Correlated with the Prognosis of Patients with Breast Cancer

**Figure 2.**
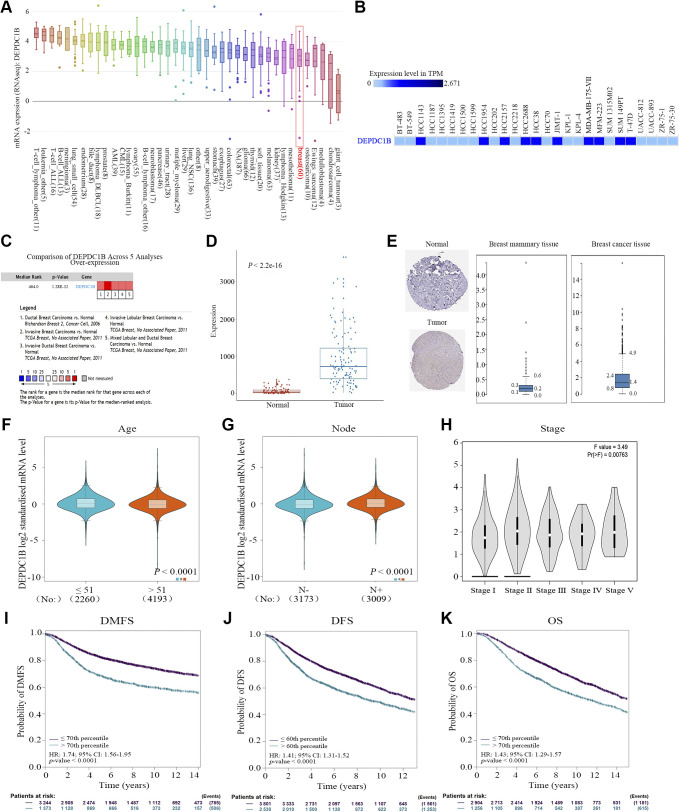
DEP domain-containing protein 1B (DEPDC1B) was highly expressed in breast cancer cells and tissues and affected the clinical prognosis of patients. *A*: DEPDC1B was expressed in various tumor cells. *B*: DEPDC1B was highly expressed in many breast cancer cells. TPM, transcript per million. *C*: DEPDC1B was highly expressed in many breast cancer tissues. *D*: DEPDC1B expression in The Cancer Genome Atlas-Breast cancer (TCGA-BRCA) cancer tissues is higher than in paracancerous tissues. *E*: DEPDC1B expression in breast cancer tissue is higher than in normal breast tissue. *F*: DEPDC1B expression was higher in breast cancer patients <51 yr old. *G*: DEPDC1B expression was higher in breast cancer patients with lymph node metastasis. *H*: DEPDC1B expression increased with increasing clinical stage. *I–K*: DEPDC1B expression is associated with shorter distant metastasis-free survival (DMFS; *I*), disease-free survival (DFS; *J*), and overall survival (OS; *K*) in breast cancer patients. HR, hazard ratio; 95% CI, 95% confidence interval.

**Figure 3.**
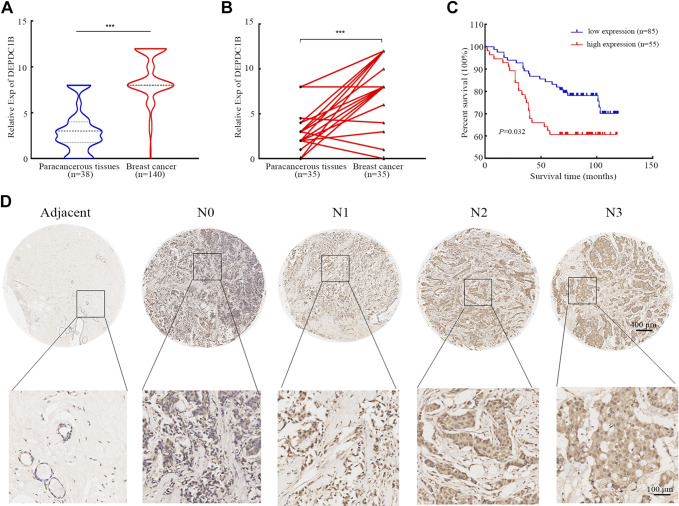
The expression of DEP domain-containing protein 1B (DEPDC1B) in breast cancer tissue microarray and its association with overall survival. *A*: DEPDC1B expression was higher in breast cancer than in paracancerous tissues. Analysis between 2 groups was conducted by unpaired Student’s *t* test. ***P < 0.0001. *B*: DEPDC1B expression was higher in paired cancer tissues than in paracancerous tissues. Analysis between 2 groups was conducted by paired *t* test. ***P < 0.0001. *C*: the overall survival (OS) time of the high-DEPDC1B expression group was shorter than that of the low-expression group. *D*: immunohistochemistry images of DEPDC1B expression in breast cancer tissues at different N stages.

**Figure 4.**
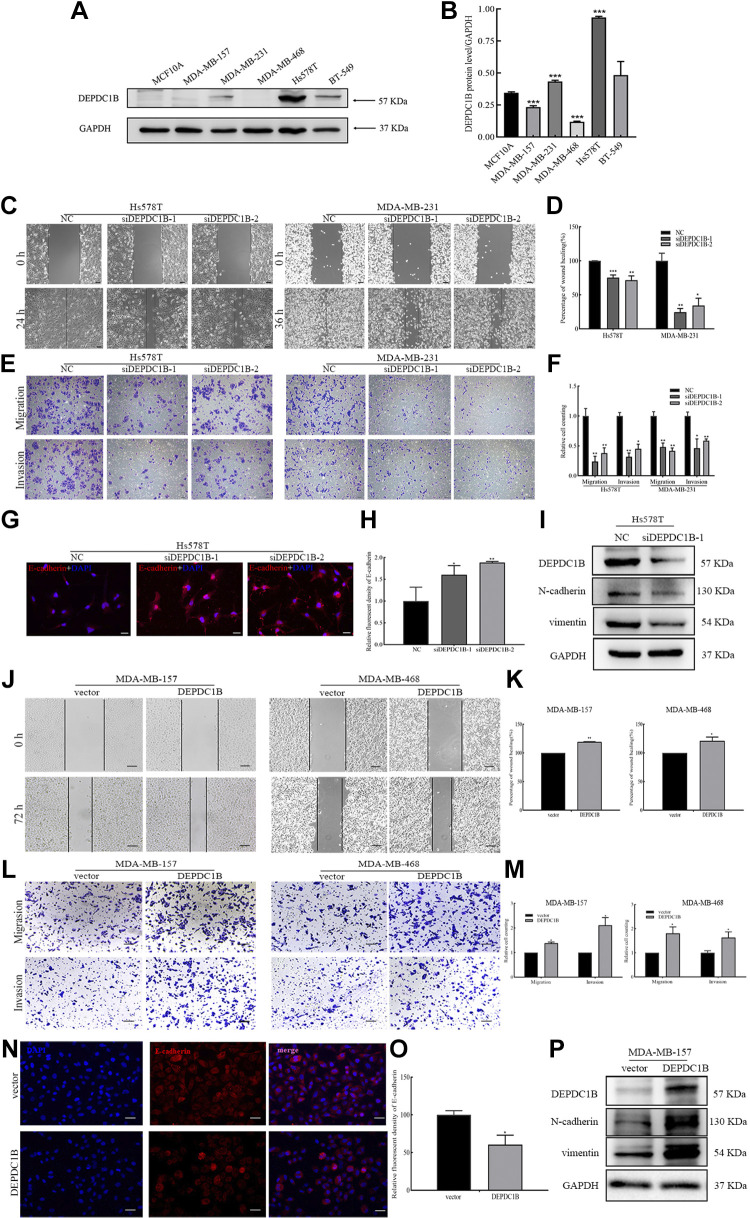
DEP domain-containing protein 1B (DEPDC1B) promotes the migration, invasion, and epithelial-mesenchymal transformation (EMT) process of breast cancer cells. *A*: DEPDC1B is highly expressed in Hs578T, MDA-MB-231, and BT-549 cells; its expression is low in MCF10A, MDA-MB-157, and MDA-MB-468 cells. *B*: the quantitative results of *A*. Analysis between multiple groups was conducted by 1-way ANOVA. ***P < 0.001. *C*: knockdown of DEPDC1B inhibits the migration of breast cancer cells. Hs578T and MDA-MB231 cells were transfected with negative control (NC) and DEPDC1B siRNA for 24 h, and the migration ability of cells was detected by scratch test. Scale bars, 100 μm. *D*: statistical analysis of *C*. Analysis between multiple groups was conducted by 1-way ANOVA. *P < 0.05; **P < 0.01; ***P < 0.001. *E*: knockdown of DEPDC1B inhibits the migration and invasion of breast cancer cells. Transwell assays were used to detect the migration and invasion ability of the cells. Scale bars, 100 μm. *F*: statistical analysis of *E*. Analysis between multiple groups was conducted by 1-way ANOVA. *P < 0.05; **P < 0.01; *G*: knockdown of DEPDC1B leads to the increase of E-cadherin. Hs578T cells were transfected with NC and DEPDC1B siRNA for 48 h, and the expression of E-cadherin was detected by immunofluorescence. Scale bars, 20 μm. *H*: statistical analysis of *G*. Analysis between multiple groups was conducted by 1-way ANOVA. *P < 0.05; **P < 0.01. *I*: knockdown of DEPDC1B results in the decrease of N-cadherin and vimentin. Western blotting was used to detect the expression of DEPDC1B, N-cadherin, and vimentin. *J*: overexpression of DEPDC1B promotes the migration of breast cancer cells. MDA-MB-157 and MDA-MB-468 cells were transfected with vector and DEPDC1B overexpression plasmids for 24 h, and the migration ability of cells was detected by scratch test. Scale bars, 100 μm. *K*: statistical analysis of *J*. Analysis between 2 groups was conducted by unpaired Student’s *t* test. *P < 0.05; **P < 0.01. *L*: overexpression of DEPDC1B promotes the migration and invasion of breast cancer cells. The Transwell test was used to detect the migration and invasion ability of cells. Scale bars, 100 μm. *M*: statistical analysis of *L*. Analysis between 2 groups was conducted by unpaired Student’s *t* test. *P < 0.05. *N*: overexpression of DEPDC1B leads to the decrease of E-cadherin. MDA-MB-157 cells were transfected with vector and DEPDC1B overexpression plasmids for 48 h, and the expression of E-cadherin was detected by immunofluorescence assay. Scale bars, 20 μm. *O*: statistical analysis of *N*. Analysis between 2 groups was conducted by unpaired Student’s *t* test. *P < 0.05. *P*: overexpression of DEPDC1B results in the increase of N-cadherin and vimentin. Western blotting was used to detect the expression of DEPDC1B, N-cadherin, and vimentin. Error bars represent the mean ± SD from 3 independent experiments.

Through online tool analysis, we found that DEPDC1B is highly expressed in a variety of tumor cells (Fig. 2*A*), including a variety of breast cancer cell lines (Fig. 2*B*), which is consistent with our cell test results (see Fig. 4, *A* and *B*). Compared with normal tissues, DEPDC1B was highly expressed in breast cancer tissues (Fig. 2, *C*–*E*), which was consistent with the results of the tissue microarray test (Fig. 3, *A* and *B*). In addition, we further analyzed the correlation between DEPDC1B expression and clinical factors of breast cancer. The results showed that DEPDC1B was correlated with age, lymph node metastasis, and clinical stage (Fig. 2, *F*–*H*). At the same time, high DEPDC1B expression was associated with shorter distant metastasis-free survival (DMFS), disease-free survival (DFS), and OS in breast cancer patients (Fig. 2, *I–K*). However, our tissue chip analysis showed that DEPDC1B expression was only correlated with lymph node metastasis (χ^2^ = 9.098, *P* = 0.028) (Table 1, Fig. 3*D*) and positively correlated with poor prognosis (χ^2^ = 5.487, *P* = 0.019) (Table 1) and resulted in poor OS (Fig. 3*C*). More noteworthy is that univariate and multivariate Cox regression analyses showed that high expression of DEPDC1B was an independent risk factor for poor prognosis in breast cancer patients (Table 2, Table 3).

**Table 1.** Correlation of DEPDC1B expression with the clinicopathological characteristics of breast cancer patients

Characteristics	*N*	DEPDC1B Expression		Chi-Square Test	
		Low or none, no. of cases (*n*)	High, no. of cases (*n*)	χ^2^ Value	*P* value
Age, yr				0.015	0.901
≤50	39 (27.9%)	24 (28.2%)	15 (27.3%)		
>50	101 (72.1%)	61 (71.8%)	40 (72.7%)		
Tumor size				3.712	0.054
<5	113 (80.7%)	73 (85.9%)	40 (72.7%)		
≥5	27 (19.3%)	12 (14.1%)	15 (27.3%)		
T classification				0.146	0.986
T_1_	35 (25.0%)	22 (25.9%)	13 (23.6%)		
T_2_	86 (61.4%)	52 (61.2%)	34 (61.8%)		
T_3_	14 (10.0%)	8 (9.4%)	6 (10.9%)		
T_4_	5 (3.6%)	3 (3.5%)	2 (3.6%)		
N classification				9.098	0.028*
N_0_	70 (50.0%)	44 (51.8%)	26 (47.3%)		
N_1_	31 (22.1%)	23 (27.1%)	8 (14.5%)		
N_2_	18 (12.9%)	11 (12.9%)	7 (12.7%)		
N_3_	21 (15.0%)	7 (8.2%)	14 (25.5%)		
Clinical stage				2.374	0.305
TNM_1_	20 (14.3%)	13 (15.3%)	7 (12.7%)		
TNM_2_	77 (55.0%)	50 (58.8%)	27 (49.1%)		
TNM_3_	43 (30.7%)	22 (25.9%)	21 (38.2%)		
Pathology grade				0.055	0.814
II	67 (47.9%)	40 (47.1%)	27 (49.1%)		
III	73 (52.1%)	45 (52.9%)	28 (50.9%)		
Survival status (at follow-up)				5.487	0.019*
Alive	95 (67.9%)	64 (75.3%)	31 (56.4%)		
Death	45 (32.1%)	21 (24.7%)	24 (43.6%)		

*N* represents number of subjects. *P < 0.05. DEPDC1B, DEP domain-containing protein 1B.

**Table 2.** Univariate analysis for overall survival (Cox proportional hazards regression model)

Variable	No.	HR	95% CI	*P* Value
DEPDC1B expression		1.885	1.023–3.475	0.042*
Low expression	85			
High expression	55			
Age, yr		1.600	0.850–3.010	0.145
≤50	39			
>50	101			
Tumor size		2.443	1.259–4.743	0.008**
<5	113			
≥5	27			
T classification		3.697	1.835–7.449	0.000***
T_1_ + T_2_	122			
T_3_ + T_4_	18			
N classification		1.404	0.763–2.586	0.276
N_0_	71			
N_1_ + N_2_ + N_3_	69			

*P < 0.05; **P < 0.01; ***P < 0.001. DEPDC1B, DEP domain-containing protein 1B; HR, hazard ratio; 95% CI, 95% confidence interval.

**Table 3.** Multivariate analysis for overall survival (Cox proportional hazards regression model)

Variable	No.	HR	95% CI	*P* Value
DEPDC1B expression		2.336	1.032–4.216	0.033*
Low expression	85			
High expression	55			
Age, yr		0.667	0.352–1.262	0.213
≤50	39			
>50	101			
T classification		3.559	1.760–7.196	0.000***
T_1_ + T_2_	122			
T_3_ + T_4_	18			
N classification		1.323	0.715–2.447	0.373
N_0_	71			
N_1_ + N_2_ + N_3_	69			

*P < 0.05; ***P < 0.001. DEPDC1B, DEP domain-containing protein 1B; HR, hazard ratio; 95% CI, 95% confidence interval.

In summary, these data show that DEPDC1B was upregulated in breast cancer tissues and cells and high DEPDC1B expression was highly predictive of poor prognosis in breast cancer patients.

### DEPDC1B Promotes the Invasion, Migration, and EMT of Breast Cancer Cells in Vivo and in Vitro

To further understand the role of DEPDC1B in breast cancer, we performed GSEA. The results show that DEPDC1B is closely related to the regulation of the cell cycle, cell proliferation, and metastasis (Supplemental Fig. S3). Previous studies have shown that DEPDC1B regulates the cell cycle in multiple cells and proliferation in HEK-293T cells (10, 39). Here, we studied the effect of DEPDC1B on the invasion and migration of breast cancer cells. Considering the high expression of DEPDC1B in Hs578T and MDA-MB-231 cells, the cell lines were selected for the siRNA interference expression experiment. Similarly, the expression of DEPDC1B is low in MDA-MB-157 and MDA-MB-468 cells, and they were used in the plasmid transfection overexpression experiment (Fig. 4, *A* and *B*). siRNA-DEPDC1B was transfected into Hs578T and MDA-MB231 cells (Supplemental Fig. S4, *A*–*D*). Scratch tests and Transwell assays showed that interference with DEPDC1B expression significantly inhibited the migration and invasion ability of breast cancer cells (Fig. 4, *C*–*F*). It is well known that arrest of the EMT process usually leads to the weakening of cell migration and invasion. Therefore, we detected EMT indicators after interfering with DEPDC1B expression in breast cancer cells. The results showed that after DEPDC1B downregulation the expression of E-cadherin increased and that of N-cadherin and vimentin was downregulated (Fig. 4, *G–I*). To fully prove that DEPDC1B expression can affect the migration and invasion ability of breast cancer cells, DEPDC1B overexpression plasmids were transfected into MDA-MB-157 and MDA-MB-468 cells (Supplemental Fig. S4, *E* and *F*). In contrast, DEPDC1B overexpression significantly promoted the migration and invasion of breast cancer cells and promoted the EMT process in cells (Fig. 4, *J–P*).

**Figure 5.**
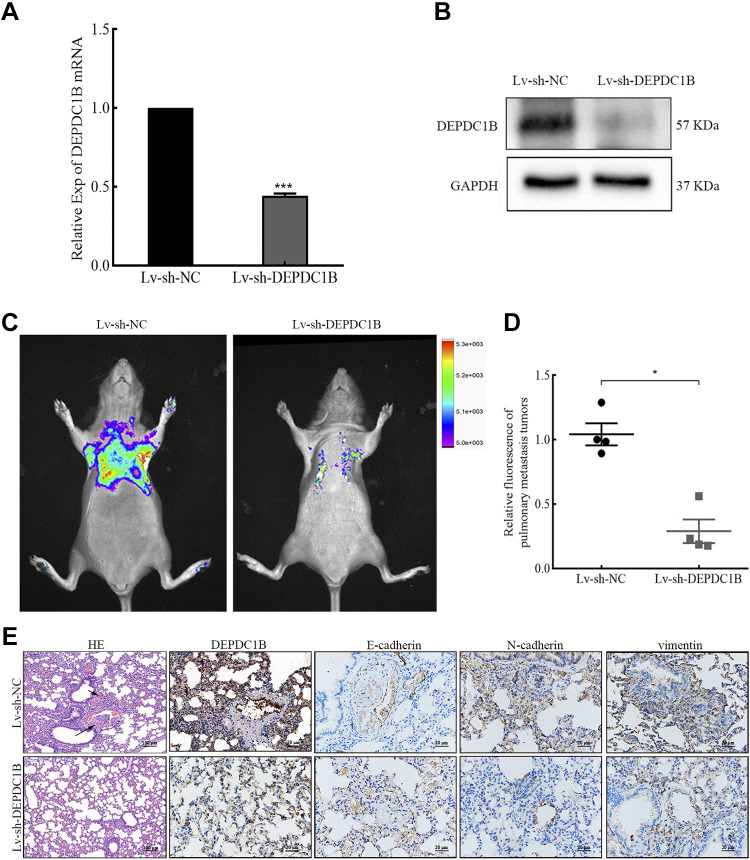
Decreased DEP domain-containing protein 1B (DEPDC1B) expression inhibited breast cancer cell migration in vivo. *A*: the results of qRT-PCR analysis confirmed DEPDC1B knockdown efficiency in steady cell lines. Analysis between 2 groups was conducted by unpaired Student’s *t* test. ***P < 0.001. The measurement data are expressed as means ± SD. Lv, lentivirus; NC, negative control. *B*: the results of Western blot analysis confirmed DEPDC1B knockdown efficiency in steady cell lines. *C*: lung metastasis model. The fluorescence of lung metastases in the Hs578T-GFP Lv-sh-DEPDC1B group was significantly weaker than that in the Lv-sh-NC group, and the fluorescence intensity was statistically analyzed (*D*). Analysis between 2 groups was conducted by unpaired Student’s *t* test. *P < 0.05. The measurement data are expressed as means ± SD. *E*: hematoxylin and eosin (HE) staining results showed the tumor nests of lung metastases. Scale bars, 100 µm. Immunohistochemistry (IHC) was used to detect the expression of DEPDC1B, E-cadherin, N-cadherin, and vimentin. Scale bars, 20 µm.

We further explored the effect of interference with DEPDC1B expression on tumor metastasis in vivo. Hs578T-Lv-sh-DEPDC1B cells were inoculated into female BALB/c-nude mice (Fig. 5, *A* and *B*). As expected, interference with DEPDC1B expression hindered tumor metastasis (Fig. 5, *C–E*). In summary, these results show that interfering with DEPDC1B expression blocks the EMT process and inhibits the migration and invasion of breast cancer cells.

### DEPDC1B in Breast Cancer Cells Activates the wnt/β-Catenin Signaling Pathway

Since previous studies have shown that DEPDC1B promotes angiogenesis and metastasis of melanoma by competing with CDC16 to promote SCUBE3 secretion (20), we investigated whether a similar phenomenon exists in breast cancer. For this, we carried out correlation analysis and found that there is a positive correlation between DEPDC1B and SCUBE3 (*R* = 0.062, *P* = 0.042), on transcription level (Supplemental Fig. S5*A*). Further analysis of breast cancer data in TCGA showed that there was no difference in SCUBE3 expression between breast cancer and paracancerous/normal tissues (Supplemental Fig. S5*B*), and there was no SCUBE3 in 364 DEGs (Supplemental Table S2). The SCUBE3 expression does not affect the survival of breast cancer patients (Supplemental Fig. S5*C*). What is more noteworthy is that there is no change of SCUBE3 expression in interference or overexpression of DEPDC1B in breast cancer cells in vitro (Supplemental Fig. S5, *D* and *E*). To sum up, it is suggested that the role of DEPDC1B in promoting breast cancer metastasis is not achieved by regulating SCUBE3.

**Figure 6.**
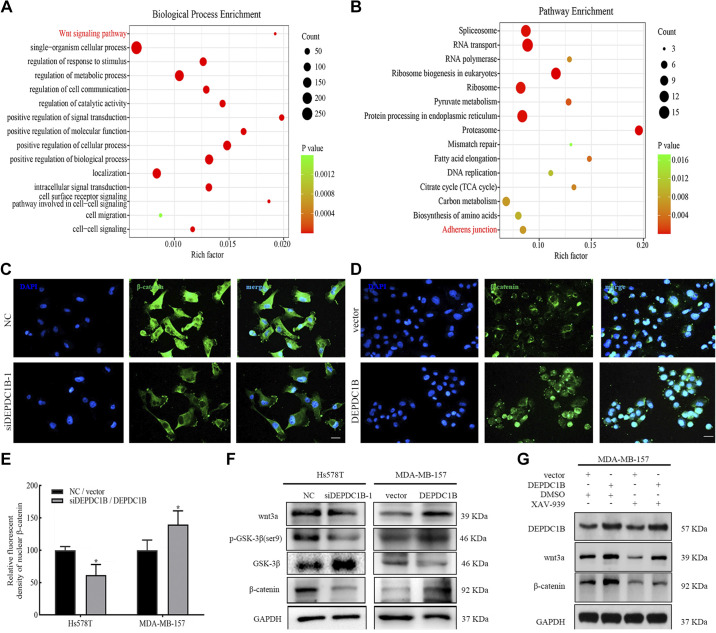
The expression of DEP domain-containing protein 1B (DEPDC1B) can activate the wnt/β-catenin signaling pathway. *A*: functional enrichment analysis of the results of protein spectrum identification. *B*: the results of KEGG analysis showed that the signal pathway related to the DEPDC1B interacting proteins. *C* and *D*: DEPDC1B is involved in the nuclear translocation of β-catenin. Hs578T cells (*C*) were transfected with negative control (NC) and DEPDC1B siRNA, MDA-MB-157 cells (*D*) were transfected with vector and DEPDC1B overexpression plasmids, and the expression of β-catenin was detected by immunofluorescence after 48 h. Scale bars, 20 μm. *E*: the results of the statistical analysis of *C* and *D*. Analysis between 2 groups was conducted by unpaired Student’s *t* test. *P < 0.05. *F*: the expression levels of wnt3a, phospho (p-)GSK-3β (ser9), GSK-3β, and β-catenin were detected by Western blot, and GAPDH was used as the internal control. *G*: Western blot results of the expression of DEPDC1B, wnt3a, and β-catenin in MDA-MB-157 cells transfected with DEPDC1B overexpression plasmid alone or in combination with XAV-939, with GAPDH as the internal control.

To explore how DEPDC1B promotes the migration and invasion of breast cancer cells, we carried out protein mass spectrometry analysis and identified 414 binding proteins specific to DEPDC1B (Supplemental Table S4). The enrichment analysis showed that they were significantly correlated with the wnt signaling pathway and adherens junctions (Fig. 6, *A* and *B*). It is well known that the wnt/β-catenin signaling pathway is one of the pathways that regulate EMT, and it is also involved in the occurrence and development of breast cancer (40, 41). The adherens junction pathway contains *CTNNB1* (gene encoding β-catenin protein) (Supplemental Table S5), which further supports that DEPDC1B may participate in the wnt/β-catenin signaling pathway in breast cancer cells. When DEPDC1B expression was silenced in Hs578T cells, β-catenin was mainly localized in the cytoplasm and significantly decreased in the nucleus (Fig. 6, *C* and *E*). At the same time, after interference with DEPDC1B expression, wnt3a, p-GSK-3β (ser9), and β-catenin expression were decreased, whereas GSK-3β expression increased. The opposite result was obtained after overexpression of DEPDC1B (Fig. 6, *D–F*). In further experiments, after cells were treated with XAV-939, an inhibitor of the wnt/β-catenin signaling pathway, combined with the DEPDC1B overexpression plasmid, the protein levels of wnt3a and β-catenin upregulated by DEPDC1B overexpression decreased (Fig. 6*G* and Supplemental Fig. S6). In summary, these results suggest that the activity of the wnt/β-catenin signaling pathway is inhibited after DEPDC1B interference in breast cancer cells.

### DEPDC1B Mediates the Ubiquitin Level of β-Catenin by Binding with USP5 and β-Catenin

**Figure 7.**
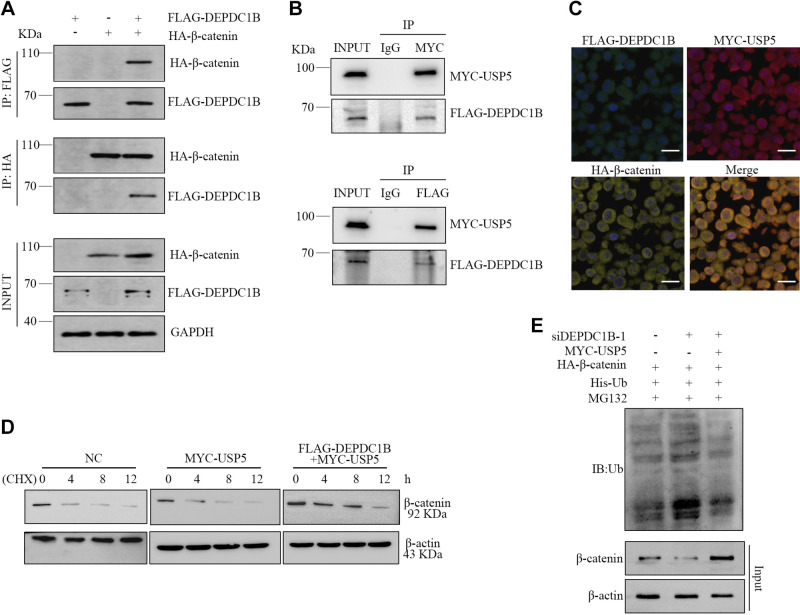
DEP domain-containing protein 1B (DEPDC1B) mediates the deubiquitination of β-catenin by ubiquitin-specific protease 5 (USP5). *A*: coimmunoprecipitation (Co-IP) results show that DEPDC1B interacts with β-catenin in breast cancer cells. MDA-MB-157 cells were transfected with FLAG-DEPDC1B and HA-β-catenin alone or in combination, and Co-IP was used to detect DEPDC1B and β-catenin. *B*: Co-IP results show that DEPDC1B interacts with USP5 in breast cancer cells. MDA-MB-157 cells were cotransfected with FLAG-DEPDC1B and MYC-USP5 plasmids, and DEPDC1B and USP5 were detected by Co-IP. *C*: DEPDC1B, USP5, and β-catenin were colocalized in breast cancer cells by detection through transfection of DEPDC1B, USP5, and β-catenin in MDA-MB-157 cells. Scale bars, 60 μm. *D*: overexpression of DEPDC1B weakens the effect of USP5 on β-catenin protein degradation level. After MDA-MB-157 cells were transfected with USP5 alone or in combination with the DEPDC1B overexpression plasmid and treated with cycloheximide (CHX) (0, 4, 8, 12 h), β-catenin was detected by Western blotting, and β-actin was used as an internal control. NC, negative control. *E*: knockdown of DEPDC1B promotes the ubiquitination of β-catenin, and combining overexpression of USP5 decreases the ubiquitination of β-catenin in breast cancer cells. MDA-MB-157 cells were treated with DEPDC1B siRNA combined with MYC-USP5, HA-β-catenin, and His-Ub and then treated with MG132 to prepare cell extracts. Anti-His was used for immunoblot (IB) analysis.

Considering that β-catenin is the core factor of the classical wnt signaling pathway, studies have shown that in breast cancer cells panobinostat inhibits the activity of the wnt/β-catenin signaling pathway by upregulating the β-catenin ubiquitination level (42). USP5 is a common cysteine deubiquitin enzyme that is highly expressed in many cancers, including breast cancer. In non-small-cell lung cancer and trophoblast cells, USP5 can deubiquitinate and stabilize β-catenin and activate the wnt/β-catenin signaling pathway (43–45). It is worth noting that our protein spectrum analysis results include both USP5 and β-catenin, indicating that there might be an interaction between DEPDC1B and both. Therefore, we hypothesized that in breast cancer cells DEPDC1B inhibits the ubiquitination of β-catenin by binding to USP5 and β-catenin. We first examined the interaction between DEPDC1B and USP5 and between DEPDC1B and β-catenin. Coimmunoprecipitation (Co-IP) results showed that there was an interaction relationship between DEPDC1B and β-catenin (Fig. 7*A*) and between DEPDC1B and USP5 (Fig. 7*B*) in breast cancer cells. Immunofluorescence results showed that DEPDC1B, USP5, and β-catenin were colocalized in breast cancer cells (Fig. 7*C*). Furthermore, the results showed that the β-catenin protein level decreased with increasing cycloheximide (CHX) treatment time, and the β-catenin protein degradation rate in the DEPDC1B plasmid combined with USP5 plasmid transfection group was significantly lower than that in the other two groups (Fig. 7*D*). Further detection of the ubiquitination level of β-catenin showed that the interfering DEPDC1B expression group was higher than that of the control group, but this increased ubiquitination level could be restored by cotransfection of the USP5 plasmid (Fig. 7*E*). In summary, we can preliminarily confirm that DEPDC1B mediates the ubiquitin level of β-catenin by binding with USP5 and β-catenin in breast cancer cells.

## DISCUSSION

Although the level of medical knowledge has greatly improved, with the extension of human life expectancy and lifestyle changes, cancer is still the leading cause of death in the world. Among cancers, breast cancer is the most common malignant disease in women, it ranks first in terms of morbidity or mortality (1), and the age of onset is gradually getting younger (46). Age, family history, menstrual status, lifestyle, and ionizing radiation are all risk factors for breast cancer (3, 47). However, because breast cancer is a highly heterogeneous disease, elucidating its pathogenesis is still a difficult point in the scientific community (2). Although the popularization of the concept of early screening has greatly improved the early diagnosis rate of breast cancer patients, most patients still have distant metastasis and are unable to benefit clinically because of the lack of effective individualized treatment, which may be related to the lack of effective early diagnostic markers and accurate treatment targets (3, 4, 48). Therefore, there is an urgent need to find new diagnostic and prognostic markers, to develop more effective diagnosis and treatment methods, and to develop new strategies to evaluate prognosis to improve the early diagnosis rate of patients and improve the overall treatment efficiency and prognosis.

We found through bioinformatics analysis that DEPDC1B is the key DEG affecting the occurrence and development of breast cancer. DEPDC1B is highly expressed in breast cancer, which is closely related to poor prognosis. Functional enrichment analysis showed that DEPDC1B was closely related to invasion and migration. Further tissue microarray analysis showed that the high expression of DEPDC1B was an independent risk factor for poor prognosis in breast cancer patients. Previous studies have shown that DEPDC1B is highly expressed in a variety of cancers, and its role is mainly related to regulating the cell cycle, promoting cell proliferation, invasion, and metastasis, and inhibiting apoptosis (49–51). In addition, DEPDC1B accumulates in the G2 phase of the cell cycle in multiple cells and regulates cell mitotic kinetics through the DEPDC1B/RhoA/PTPRF axis to promote the cell transition from G2 phase to M phase (39). However, it is not known whether DEPDC1B plays a carcinogenic role in breast cancer by affecting invasion and migration. Here, we proved that interference with DEPDC1B expression can significantly inhibit the metastasis of breast cancer cells in vivo and in vitro. In summary, our study further confirmed the tumor-promoting effect of DEPDC1B on breast cancer, which may be a potential therapeutic target for breast cancer.

It is well known that EMT often occurs during tumor cell migration (52). EMT refers to the process of transformation from epithelial cells to mesenchymal cells, which is mainly characterized by a decrease in E-cadherin and an increase in N-cadherin and vimentin (53). However, the kinetic and molecular mechanisms of EMT-related cancer heterogeneity are unclear. Understanding the mechanism of EMT regulation will be conducive to the development of new therapeutic strategies to prevent cancer progression and metastasis. Our in vitro and in vivo results show that high DEPDC1B expression promotes the EMT process in breast cancer cells. One of the classical pathways regulating the EMT process is the Wnt/β-catenin signaling pathway (40). Breast cancer is the first cancer reported to be associated with this signaling pathway (41). Wnt signaling is closely related to breast cancer proliferation, metastasis, immune microenvironment regulation, dry maintenance, treatment resistance, and so on (41). A variety of Wnt signal inhibitors targeting different targets have been developed, but they have not been approved for clinical application (41). Through functional enrichment analysis of DEPDC1B-interacting proteins, we found that they are highly enriched in the wnt signaling pathway and are closely related to adhesion junction function. Our results confirmed that DEPDC1B activated the wnt/β-catenin signaling pathway in breast cancer cells. In addition, further exploration of the protein spectrum results showed that β-catenin was highly enriched in adhesion junction function.

β-Catenin, encoded by *CTNNB1*, is the core factor of the classical wnt signaling pathway. It binds to the cytoplasmic tail of E-cadherin and plays a role in cell-cell adhesion. In the absence of wnt signal stimulation, β-catenin in the cytoplasm is phosphorylated by a destructive complex composed of APC, Axin, GSK3β, CK1α, PP2A, and WTX and then ubiquitinated by SCF^β-TRCP^, a protein composed of Skp1, Cullin1, and F-box (41). The specific process is that CK1α induces phosphorylation of the S45 site and then GSK3β induces phosphorylation of the S33, S37, and T41 residues, while phosphorylation of S33 and S37 provides ubiquitination sites for SCF^β-TRCP^, induces ubiquitination of β-catenin, and is finally recognized and degraded by the proteasome (41). In contrast, when cells are stimulated by wnt signaling, the destruction complex is inactivated, β-catenin cannot be phosphorylated, and less β-catenin can be recognized and degraded by ubiquitin enzymes, accumulate in the cytoplasm, be transported to the nucleus by APC, and bind to TCF/LEF transcription, activating the expression of downstream target genes (54). It has been reported that in breast cancer the ubiquitin level of β-catenin can be upregulated by panobinostat, thus inhibiting the growth and metastasis of breast cancer. The mechanism is that panobinostat increases the level of APCL expression in the wnt/β-catenin signaling pathway (42). The regulation of ubiquitin-mediated degradation of β-catenin includes the classical pathways mentioned above, as well as other regulatory pathways. In lung cancer cells, the ubiquitination level of β-catenin can be increased by FBXW2, whereas cell migration and invasion are significantly inhibited, and the dominant regulatory mechanism is the accumulation of β-catenin^ser552^ caused by the EGFR-AKT signal pathway and ubiquitin degradation by FBXW2 (55). Here, we indicate that there is an interaction between DEPDC1B and β-catenin in breast cancer cells, but it is not clear whether DEPDC1B is also involved in the ubiquitin process.

Previous studies have shown that β-catenin can be stabilized by USP5 through deubiquitination, activate the wnt/β-catenin signaling pathway, and play a related pathogenic role (44, 45). It has also been reported that high USP5 expression in breast cancer leads to poor clinical prognosis, and its molecular mechanism is related to the promotion of breast cancer cell proliferation, migration, and invasion by stabilizing the HIF2α protein through deubiquitination activity (56). Ubiquitin-specific proteinase 5 (USP5), also known as ubiquitin isopeptidase T (ISOT), is a cysteine deubiquitin enzyme that belongs to the USP family (43). Our protein spectrum analysis results suggest that USP5 and DEPDC1B are interacting proteins, which has also been verified through experiments. In addition, we also proved that DEPDC1B, USP5, and β-catenin colocalize in breast cancer cells and that DEPDC1B promotes the deubiquitination of β-catenin by USP5.

Our study reveals for the first time that DEPDC1B may be a potential therapeutic target for breast cancer patients. However, there are still limitations to this study. First, mechanistically, how DEPDC1B, USP5, and β-catenin interact, the location of the binding sites, how DEPDC1B mediates the deubiquitination of β-catenin by USP5, and whether DEPDC1B has enzyme activity remain to be further explored. Second, our results need to be supported by further clinical studies.

### Conclusions

**Figure 8.**
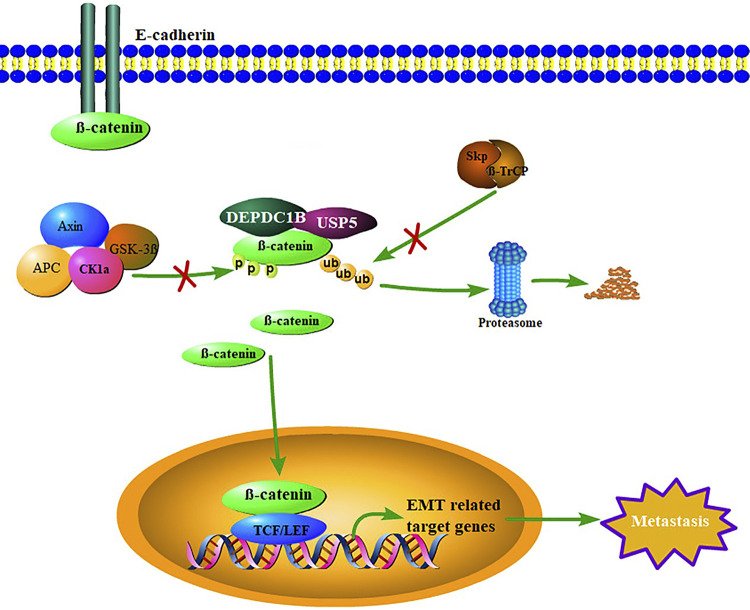
Schematic map of the DEP domain-containing protein 1B (DEPDC1B) regulatory pathway in breast cancer cells. In cancer cells, some β-catenin binds to E-cadherin to maintain cell adhesion, and some β-catenin can be phosphorylated by a degradation complex composed of Axin, APC, CK1a, and GSK-3β, ubiquitinated by Skp/β-TrCP, and then degraded by the proteasome. Some undegraded β-catenin translocates into the nucleus, binds to TCF/LEF, activates target gene transcription and expression, and plays a corresponding function. In breast cancer cells, DEPDC1B enhances the deubiquitination of β-catenin by ubiquitin-specific protease 5 (USP5) through interaction with USP5 and β-catenin, further stabilizing β-catenin protein, promoting its translocation into the nucleus, activating the transcription of epithelial-mesenchymal transformation (EMT)-related target genes, and ultimately promoting the metastasis of breast cancer cells.

Our results show that DEPDC1B is upregulated in breast cancer tissues and cells, which promotes cell metastasis by promoting the EMT process of cells. In addition, we also found that high DEPDC1B expression is an independent risk factor for breast cancer patients, leading to worse OS. It is worth noting that our study proved for the first time that there is a high correlation between DEPDC1B and breast cancer cell metastasis. Finally, we found that in breast cancer cells DEPDC1B mediates the deubiquitination of β-catenin by USP5, stabilizes the β-catenin protein level, promotes nuclear translocation, activates the wnt/β-catenin signaling pathway, and ultimately promotes the transfer of breast cancer cells (Fig. 8). Therefore, our findings provide new insights into the carcinogenic role of DEPDC1B in breast cancer, suggesting that DEPDC1B may be a reliable prognostic factor and a potential therapeutic target for breast cancer.

## ETHICAL APPROVALS

Animal experiments were reviewed and approved by the Institutional Animal Care and Use Committee of Wuhan University (No. SQ20200266).

## DATA AVAILABILITY

The technical appendix, statistical code, and dataset are available from the corresponding author at yfzhouwhu@163.com. Consent was given for data sharing by participants.

## SUPPLEMENTAL MATERIAL

10.6084/m9.figshare.23816421 Supplemental Tables S1–S5 and Supplemental Figs. S1–S5: https://doi.org/10.6084/m9.figshare.23816421.

## GRANTS

This work was supported by the National Natural Science Fund of China (No. 81472799) and the Fundamental Research Funds for the Central Universities (Grant No. 2042022kf1141).

## DISCLOSURES

No conflicts of interest, financial or otherwise, are declared by the authors.

## AUTHOR CONTRIBUTIONS

Y.Z. conceived and designed research; Q.W., L.X., and X.Y. performed experiments; Q.W., F.C., N.Y., and L.X. analyzed data; Q.W., F.C., N.Y., and L.X. interpreted results of experiments; Q.W. and F.C. prepared figures; Q.W., N.Y., and L.X. drafted manuscript; M.W. and Y.Z. edited and revised manuscript.
